# Functional Status of Patients with Long-Term Mechanical Left Ventricular Assist Device Support in Relation to Physical Activity

**DOI:** 10.3390/jcm15124602

**Published:** 2026-06-13

**Authors:** Julia Zuzanna Bura, Zuzanna Strząska-Kliś, Radosław Wilimski, Mariusz Kuśmierczyk, Daniel Karaszewski

**Affiliations:** 1 Rehabilitation Department, Józef Piłsudski University of Physical Education in Warsaw, 00-968 Warsaw, Poland; jb74955@stud.awf.edu.pl; 2 Department of Cardiac, Thoracic and Transplant Surgery, Medical University of Warsaw, 02-097 Warsaw, Poland; zuzanna.strzaska-klis@wum.edu.pl (Z.S.-K.); prof.kusmierczyk@gmail.com (M.K.)

**Keywords:** physical activity, LVAD, heart failure, functional status, rehabilitation

## Abstract

**Background/Objectives**: Advanced heart failure is associated with reduced functional capacity and impaired quality of life. Left ventricular assist devices (LVADs) are increasingly used as a long-term treatment option in patients with end-stage heart failure. Despite improvements in hemodynamic function after LVAD implantation, many patients continue to experience limitations in daily functioning. The aim of this study was to evaluate the relationship between physical activity and functional status in patients with LVAD support. **Methods**: This study included 262 adult participants divided into four groups according to LVAD support and declared physical activity. Functional status and quality of life were assessed using the Short Form-36 Health Survey (SF-36) and the Minnesota Living with Heart Failure Questionnaire (MLHFQ). **Results**: Significant differences were observed between the analyzed groups in both SF-36 and MLHFQ scores. Physically active patients with LVAD achieved the most favorable results, indicating a better functional status and lower symptom burden, whereas inactive individuals demonstrated poorer outcomes. Significant correlations were found between physical activity and selected aspects of daily functioning, including walking, climbing stairs, performing household activities, and carrying groceries. Higher levels of physical activity were associated with better quality of life and fewer functional limitations. **Conclusions**: Physical activity may positively influence functional status and quality of life in patients with LVAD support. The findings suggest that regular physical activity should be considered an important component of rehabilitation and long-term management in patients with advanced heart failure treated with LVAD therapy.

## 1. Introduction

Heart failure is a complex clinical syndrome characterized by symptoms and/or signs resulting from structural or functional abnormalities of the heart, often accompanied by increased natriuretic peptide levels or signs of fluid retention [1]. It represents a significant global health challenge, affecting approximately 1–3% of the adult population. Although the incidence of the disease has slightly declined over time, its overall prevalence continues to rise, largely due to population ageing and improved survival due to advances in treatment. Despite therapeutic progress, the prognosis of heart failure remains unfavorable, with mortality rates estimated at approximately 15–30% within the first year and up to 50–75% within five years. Furthermore, the condition imposes a significant burden on healthcare systems, leading to frequent hospitalizations and substantial healthcare expenditures [2]. Maintaining quality of life and physical functioning is considered equally important as prolonging survival in patients with chronic and progressive diseases. In heart failure, both quality of life and exercise tolerance are markedly impaired compared to the general population as well as to a broad spectrum of physical and psychological symptoms, including dyspnea, fatigue, fluid accumulation, sleep disturbances, and depression. As a consequence, these symptoms significantly restrict patients’ ability to engage in everyday physical and social activities [3].

Despite advances in pharmacological treatment and the use of cardiac assist devices, a subset of patients progresses to advanced heart failure, representing the most severe stage of the disease. According to the current guidelines of the European Society of Cardiology, advanced heart failure is characterized by persistent severe symptoms, typically corresponding to New York Heart Association (NYHA) class III or IV, despite optimal guideline-directed medical therapy. These patients frequently experience recurrent episodes of decompensation requiring hospitalization, exhibit severe cardiac dysfunction, and have markedly reduced exercise capacity [4,5]. It is estimated that patients with advanced heart failure account for approximately 5–10% of the overall heart failure population [6].

The contemporary classification of heart failure takes into account both the structural and clinical aspects of the disease. According to the guidelines of the European Society of Cardiology, the basic division is based on the value of the left ventricular ejection fraction, distinguishing heart failure with a reduced ejection fraction (HFrEF, LVEF ≤ 40%), mildly reduced ejection fraction (HFmrEF, LVEF 41–49%), and preserved ejection fraction (HFpEF, LVEF ≥ 50%). This classification reflects pathophysiological differences and serves as an important basis for selecting appropriate therapy. In clinical practice, however, the assessment of symptom severity plays an important role, and it is performed using the New York Heart Association Functional Classification (NYHA). This classification allows for determining the degree of limitation in physical activity tolerance from class I, where daily activity does not cause symptoms, to class IV, characterized by symptoms present even at rest [7].

Treatment of heart failure depends on its phenotype and the stage of disease progression. In patients with heart failure with a reduced ejection fraction, the most important part of therapy is modern pharmacological treatment based on several key drug classes, the combined use of which significantly reduces mortality and the rate of hospitalization. Treatment includes several approaches and should be initiated as early as possible and optimized throughout the course of patient follow-up [8,9]. In patients with heart failure with a preserved ejection fraction, therapeutic options remain relatively limited; nevertheless, the introduction of SGLT2 inhibitors has constituted a significant advancement, as this class of agents has demonstrated a favorable impact of clinical outcomes [10]. Regardless of the heart failure phenotype, selected patients may benefit from device-based therapies, including cardiac resynchronization therapy (CRT) in individuals presenting with a widened QRS complex and a reduced ejection fraction, as well as the use of an implantable cardioverter defibrillator (ICD) for the primary or secondary prevention of sudden cardiac death. In advanced stages of heart failure, it may be necessary to consider advanced therapy strategies, such as mechanical circulatory support with a left ventricular assist device (LVAD) or orthotopic heart transplantation as definitive treatment modalities [11].

According to the guidelines of the European Society of Cardiology (ESC, 2021), the European Association for Cardio-Thoracic Surgery (EACTS, 2019), and the Kassenärztliche Bundesvereinigung (KBV, 2019) regarding the diagnosis and management of heart failure, candidates for left ventricular assist device (LVAD) implantation include patients with severe heart failure persisting for at least two months despite optimal pharmacological therapy and/or device-based treatment. Eligibility is based on the presence of more than one of the following criteria: markedly reduced left ventricular ejection fraction (LVEF ≤25%) or, if cardiopulmonary exercise testing has been performed, a peak oxygen consumption <12 mL/kg/min; at least three hospitalizations within the previous year due to unexplained decompensation of heart failure; absence of severe right ventricular failure with significant tricuspid regurgitation; and progressive end-organ dysfunction including renal and/or hepatic impairment resulting from hypoperfusion, accompanied by adverse hemodynamic parameters as well as dependence on intravenous inotropic support [7,12,13,14,15].

Qualification for LVAD implantation is based on the assessment of heart failure severity using the NYHA classification and the INTERMACS profile. The INTERMACS scale includes seven clinical profiles—patients classified as INTERMACS 3–4 are most commonly considered for elective LVAD implantation, whereas those in profiles 1–2 require urgent intervention due to a life-threatening clinical status. The combined use of both classifications allows for the evaluation of functional status and supports the optimal timing of device implantation [5].

Whether a patient can receive long-term LVAD implantation depends on the presence of both absolute and relative contraindications including numerous clinical and non-clinical factors. The most significant include severe conditions, such as established liver cirrhosis, elevated Model for End-Stage Liver Disease (MELD) scores, irreversible multiorgan failure, and advanced malignancy with an expected survival of less than two years. Chronic dialysis therapy (with peritoneal dialysis considered acceptable), neuromuscular disorders limiting patient functional capacity, and psychiatric conditions impairing the ability to properly manage the device are also important. Additional contraindications are unfavorable socioeconomic circumstances, lack of caregiver support, and poor adherence to medical recommendations or inadequate cooperation with the healthcare team. Furthermore, the presence of active infective endocarditis (including device-related endocarditis with bacteremia or valvular involvement) and pregnancy are taken into account, along with recent stroke, aortic aneurysm, and others [16].

The HeartMate 3 pump is a type of left ventricular assist device (LVAD), an advanced system for mechanical left ventricular support, utilizing “Full MagLev Flow” technology based on a fully magnetically levitated rotor, which eliminates friction and enhances hemocompatibility. The system consists of an implanted pump as well as external components, including a driveline, a controller that monitors pump parameters, and two batteries providing power supply. The system is complemented by a mobile power unit, allowing the patient to function outside the hospital environment. The driveline exit site is most commonly located in the lower abdominal quadrant and requires continuous protection with a sterile dressing [17].

Implantation of the left ventricular assist device leads to significant hemodynamic changes, including an increase in cardiac output and cardiac index, as well as a reduction in pulmonary capillary wedge pressure and pulmonary vascular resistance. This results in left ventricular unloading, improved end-organ perfusion, and a reduction in congestive symptoms; however, in some patients, it may contribute to the development of right ventricular failure due to alterations in preload and afterload conditions [18]. Despite significant improvements in hemodynamic parameters following LVAD implantation, patients’ exercise capacity remains limited, primarily due to insufficient cardiac reserve, right ventricular dysfunction, and peripheral factors such as skeletal muscle dysfunction. Nevertheless, the increase in cardiac output and enhancement of end-organ perfusion contribute to improved exercise tolerance and overall functional status [19].

Physical activity is defined as any movement generated by skeletal muscle contraction that results in energy expenditure above resting levels and is a basic component of maintaining health. Functional status, in turn, refers to an individual’s ability to perform daily activities necessary to meet basic needs, fulfill social roles, and maintain health and well-being [20]. Importantly, physical activity is closely associated with the functional status, as higher levels enhance the ability to perform daily tasks, and support overall physical functioning [21].

Exercise-based cardiac rehabilitation constitutes an essential component of comprehensive management in patients with an implanted left ventricular assist device (LVAD). Available evidence indicates that appropriately prescribed physical training is safe and can be initiated as early as the postoperative period, contributing to improvements in exercise capacity and functional status. Regular physical activity has a beneficial impact on the ability to perform activities of daily living, enhances independence, and improves quality of life. However, it should be emphasized that the observed improvements are typically moderate and do not result in full normalization of exercise capacity, which is attributable to persistent hemodynamic limitations as well as peripheral factors, such as skeletal muscle dysfunction and prior deconditioning [22].

Current recommendations from international scientific societies, such as the European Association of Preventive Cardiology and the International Society for Heart and Lung Transplantation, indicate that cardiac rehabilitation constitutes an integral component of the management of patients with LVAD. Despite the absence of standardized training protocols, rehabilitation should be conducted in an individualized, gradual, and closely monitored manner, taking into account the patient’s clinical status and hemodynamic parameters. Early initiation of physical activity is recommended, initially including mobilization, breathing exercises, verticalization, and gait training, followed by the progressive introduction of endurance and resistance exercises of low to moderate intensity. Particular importance should be placed on the assessment of exercise safety, including monitoring of clinical symptoms and device parameters, proper management of the driveline, and avoidance of the Valsalva maneuver, abrupt positional changes, and excessive physical strain [22,23,24].

The development of therapies involving left ventricular assist devices (LVADs) has significantly changed the management of patients with advanced heart failure, contributing to improved survival and clinical status. Despite the growing number of studies in this population, the impact of physical activity on the functional status after LVAD implantation remains an area of ongoing research and is not yet fully elucidated. The aim of this study was to assess the impact of physical activity on the functional status of patients with advanced heart failure, with a particular emphasis on those after LVAD implantation.

## 2. Materials and Methods

Data were collected anonymously using an electronic questionnaire developed in Google Forms. Participants accessed the survey via a link distributed online. Recruitment was carried out through internet-based channels, particularly via VAD Polska support groups on social media, as well as through email distribution. Participants were selected according to specific inclusion and exclusion criteria. Table 1 presents the criteria used for qualification to this study. Ethical review and approval were waived for this study, as it involved only an anonymous questionnaire-based survey.

This study employed standardized and validated research instruments: the Short Form Health Survey (SF-36) and the MLHFQ Minnesota Living with Heart Failure Questionnaire (MLHFQ), as well as an original author-designed questionnaire used to collect clinical and demographic data.

For statistical analyses, selected components of the questionnaires directly related to physical activity and physical functioning were utilized. In the case of SF-36, the physical functioning (PF) subscale was included, encompassing items addressing limitations in performing daily activities such as walking various distances, climbing stairs, bending, lifting objects, dressing and bathing. This subscale reflects the level of physical fitness and the degree of functional limitations resulting from the health condition.

With regard to the MLHFQ, the analysis included items corresponding to the so- called physical dimension, referring to the impact of heart failure on physical activity and everyday functioning. This questionnaire is a validated, disease-specific instrument for patients with heart failure, enabling the assessment of symptom severity and their impact on quality of life, with higher scores indicating greater limitations and a poorer quality of life.

The collected material was subsequently analyzed with a focus on evaluating the impact of self-reported physical activity on patients’ functional status.

The study included 262 adult patients (≥18 years) diagnosed with advanced heart failure, classified as NYHA class III-IV. The study population was divided in two stages. In the first stage, based on the use of mechanical circulatory support, two groups were distinguished: patients with an implanted LVAD device and those without LVAD. Subsequently, a more detailed classification into four groups was performed, taking into account both the LVAD status and declared physical activity. As a result, four groups were identified: group 1—inactive patients without LVADs (n = 86), group 2—inactive patients with LVADs (n = 62), group 3—active patients without LVADs (n = 36), and group 4—active patients with LVADs (n = 78). In all patients receiving mechanical circulatory support, the implanted device was the Heart Mate 3 left ventricular assist device.

Table 2 presents the sociodemographic and clinical characteristics of the study groups, including age, sex, height, body weight, place of residence, smoking status, and telemonitoring; their comparison enables the assessment of differences between the groups in terms of selected demographic and health-related parameters.

Statistical analysis was performed using the “Statistica” program available under the academic license of the Józef Piłsudski University of Physical Education in Warsaw. Quantitative variables were presented using descriptive statistics, taking into account mean, minimum and maximum values, and standard deviation. Qualitative variables are presented in the form of numbers and percentages.

The normality of the data distribution was assessed using the Shapiro–Wilk test. Due to the lack of normality of the distribution of parts of the analyzed data, nonparametric statistical methods were used. In the Kruskal–Wallis test analysis, the “H” value indicated the test statistic. The level of statistical significance was assumed to be a *p*-value < 0.05.

If statistically significant results were obtained, post hoc analysis was performed using Dunn’s test with Bonferroni correction to identify differences between individual groups.

The U Mann–Whitney test was used to compare two independent groups. In the analysis, the “Z” value indicates the standardized test statistic, while the *p*-value indicates the level of statistical significance. The effect size was assessed using the “r”-factor.

The relationships between selected variables were assessed using Spearman’s rank correlation coefficient (ρ). The value of the coefficient “ρ” determined the direction along with the strength of the relationship between variables. The interpretation of the strength of correlation was based on Cohen’s classification, according to which values |ρ| = 0.10–0.2—weak correlation, 0.3–0.49—moderate correlation, and ≥0.50—strong correlation.

## 3. Results

### 3.1. Comparison of Four Study Groups

In the first stage of the analysis, four groups were compared: physically active and inactive individuals, both among patients with an implanted left ventricular assist device (LVAD) and those without it. Differences between groups were assessed using the Kruskal–Wallis test.

To facilitate interpretation of the obtained results, mean scores of the SF-36 Physical Functioning (PF) subscale and selected MLHFQ items related to physical activity and physical functioning were calculated for each study group. Higher SF-36 PF scores indicate better physical functioning, whereas higher MLHFQ scores reflect greater limitations associated with heart failure. Detailed results for all study groups are presented in Table 3.

The analysis revealed statistically significant differences for both the MLHFQ (H = 75.88; *p* < 0.001) and SF-36 (H = 75.98; *p* < 0.001) questionnaires. The effect size was large in both cases (r = 0.54), indicating a strong influence of group membership on functional status and quality of life.

Post hoc analysis using Dunn’s test with Bonferroni correction showed the following:

- Inactive individuals without LVADs differed significantly from all other groups: inactive with LVADs (*p* < 0.001; r ≈ 0.22), active without LVADs (*p* = 0.049; r ≈ 0.12), and active with LVADs (*p* < 0.001; r ≈ 0.22);
- Significant differences were also observed between active individuals without LVADs and active individuals with LVADs (*p* < 0.001; r ≈ 0.22).

No significant difference were found between inactive patients with LVADs and active groups (*p* > 0.05).

Similar patterns were observed for SF-36. Significant differences were found between inactive individuals without LVADs and both inactive with LVADs (*p* < 0.001; r ≈ 0.22) and active with LVADs (*p* < 0.001; r ≈ 0.22). Additionally, a significant difference was observed between active individuals without LVADs and active individuals with LVADs (*p* = 0.000855; r ≈ 0.20). Other comparisons did not reach statistical significance (*p* > 0.05).

Analysis of mean ranks indicated that the most favorable outcomes—both in terms of quality of life and functional status—were observed in physically active patients with LVAD, while the least favorable outcomes were found in inactive individuals without LVAD.

Further analysis demonstrated the following:

- In the LVAD group, physical activity significantly improved both MLHFQ (*p* < 0.001; r ≈ 0.22) and SF-36 scores (*p* = 0.000855; r ≈ 0.20);
- In patients without LVADs, the impact of physical activity was limited (SF-36: *p* = 0.073; MLHFQ: *p* = 0.049; r ≈ 0.12).

Importantly, no significant differences were observed between inactive patients with and without LVADs (MLHFQ: *p* = 0.375; SF-36: *p* = 0.113), indicating that the presence of the device alone, without accompanying physical activity, does not significantly influence functional outcomes.

### 3.2. Impact of LVADs and Clinical Factors

Subsequently, pairwise comparisons were conducted using the U Mann–Whitney test across the entire study population.

The presence of LVADs significantly differentiated both functional statuses (for SF-36: Z = 7.95 and *p* < 0.001 and for MLHFQ: Z = −7.88; *p* < 0.001). However, this effect should be interpreted in the context of overall analysis, as it is influenced by the level of physical activity.

Further analysis revealed the following:

- Comorbidities did not significantly affect SF-36 (Z = −1.51; *p* = 0.13), but significantly influenced MLHFQ (Z = 4.17; *p* < 0.001);
- Telemonitoring significantly affected both questionnaires (SF-36: Z = −7.69; *p* < 0.001; MLHFQ: Z = 6.17; *p* < 0.001);
- Smoking did not significantly influence SF-36 (Z = 1.40; *p* = 0.16), but showed a significant association with MLHFQ (Z = −2.01; *p* = 0.044).

These findings suggest that MLHFQ is more sensitive than SF-36 in detecting the impact of clinical and behavioral factors.

### 3.3. Impact of Physical Activity on Functional Status

Comparative analysis demonstrated significant differences between physically active and inactive individuals. For SF-36, the result was Z = 4.62; *p* < 0.001, with an effect size of r = 0.28, indicating a moderate effect. Similarly, for MLHFQ: Z = −5.57; *p* < 0.001; r = 0.34, also indicating a moderate effect. These results indicate that physical activity is associated with a better functional status and reduced disease-related limitations.

### 3.4. Correlation Analysis

Spearman’s rank correlation analysis revealed a significant relationship between physical activity and questionnaire outcomes.

A positive correlation was observed between physical activity and SF-36 (ρ = 0.285; *p* < 0.05), indicating an improved functional status with increasing activity levels. Conversely, a negative correlation was found between physical activity and MLHFQ (ρ = −0.343; *p* < 0.05), suggesting that higher activity levels are associated with fewer functional limitations. The strength of these relationships was classified as weak to moderate.

### 3.5. Detailed Analysis-SF-36 Items

Further analysis examined correlations between selected items from the physical functioning domain of SF-36 and MLHFQ scores. All variables demonstrated statistically significant negative correlations. Detailed results of the correlation analysis are presented in Table 4.

This results indicate that increased limitations in performing daily physical activities are associated with poorer quality of life as measured by MLHFQ.

### 3.6. Detailed Analysis-MLHFQ Items

A parallel analysis was conducted for selected MLHFQ items in relation to physical functioning assessed by SF-36. The strongest correlations were presented in Table 5.

These findings indicate that a greater symptom burden in heart failure is associated with lower levels of physical functioning.

## 4. Discussion

The studies presented in this article assessed the relationship between physical activity and functional status in patients with left ventricular assist devices (LVADs), with additional comparisons involving individuals without LVAD support.

The comparison of four study groups demonstrated statistically significant differences in both SF-36 and MLHFQ outcomes. The strongest and most favorable results were observed among physically active patients with LVADs, whereas the least favorable functional outcomes were found inactive individuals without LVAD support.

Importantly, physically active LVAD recipients achieved significantly better results in both questionnaires compared with inactive LVAD patients. In contrast, among individuals without LVAD support, the influence of physical activity was less pronounced and only partially statistically significant. This observation may indicate that physical activity has particularly high clinical importance in patients with severe heart failure requiring mechanical circulatory support. Improved exercise tolerance, enhanced peripheral muscle efficiency, and better adaptation to daily activities may explain these differences.

Another important finding was the lack of significant differences between inactive patients with and without LVAD support. This may suggest that implantation of the device alone is insufficient to substantially improve functional outcomes in the absence of accompanying physical activity. Although LVAD therapy improves hemodynamic parameters and systemic perfusion, the benefits may remain limited without active patient participation in rehabilitation and regular movement-based activity.

Correlation analyses additionally confirmed the relationship between physical activity and functional status. Higher levels of physical activity were associated with higher SF-36 scores and lower MLHFQ scores, indicating a better quality of life and fewer functional limitations. The observed correlations ranged from weak to strong, depending on the analyzed activity. Particularly strong associations were identified for activities requiring greater physical capacity, such as walking longer distances, climbing stairs, and performing household tasks. These findings emphasize the importance of maintaining mobility and endurance in daily life among LVAD patients.

The analysis of individual MLHFQ items also demonstrated that symptoms such as dyspnea, fatigue, sleep disturbances, reduced social interaction, and limitations in household activities were significantly associated with poorer physical functioning. This observation is consistent with the clinical characteristics of advanced heart failure, in which the symptom burden directly limits independence and participation in everyday activities.

These findings are consistent with previous studies indicating that cardiac rehabilitation and regular physical activity improve exercise tolerance, quality of life, and overall functional capacity in patients after LVAD implantation. Current recommendations of organizations such as the European Association of Preventive Cardiology and the International Society for Heart and Lung Transplantation emphasize the importance of individually tailored rehabilitation programs and the gradual implementation of physical activity in LVAD recipients [25].

In addition to findings derived from structured rehabilitation programs, previous studies have also emphasized the role of exercise training in improving functional outcomes in patients supported with LVADs. Kerrigan et al. demonstrated that participation in cardiac rehabilitation following LVAD implantation leads to significant improvements in functional capacity and patient-reported health status [26]. Similarly, other studies have confirmed the beneficial effects of exercise interventions on exercise tolerance and overall physical performance in this population [27].

Importantly, these observations extend beyond supervised interventions. Despite substantial hemodynamic improvement following LVAD implantation, exercise capacity often remains limited and varies considerably between patients. As highlighted in previous studies, this variability may be influenced by multiple factors, including comorbidities, peripheral conditioning, and overall rehabilitation status. In this context, physical activity may represent a key modifiable factor contributing to a better functional performance in LVAD recipients [28].

The literature also highlights a growing interest in intensifying cardiac rehabilitation. Karaszewski et al. demonstrated that the use of a more intensive rehabilitation model in patients following cardiac surgery is a safe approach and is associated with better functional outcomes compared to the standard approach. These results indicate that appropriately managed physical activity can be a significant factor in improving patients’ functional status [29].

In the context of our own findings, this suggests the need for individualized planning of the rehabilitation process, taking into account the patient’s exercise capacity and clinical condition. It seems important to aim for the highest possible, yet safe, training loads that the patient can tolerate without the risk of complications. Such an approach may help optimize treatment outcomes and improve physical functioning.

Several mechanisms may explain the beneficial effects of physical activity observed in the present study. Regular exercise may improve skeletal muscle perfusion, endothelial function, respiratory efficiency, and peripheral oxygen utilization. Furthermore, increased physical activity may reduce deconditioning and support psychological well-being, social functioning, and patient independence.

A limitation of the study was the inability to fully control the participants’ private living conditions and factors that could influence their daily functioning and level of physical activity, such as motivation, support from family and friends, financial and architectural constraints, etc. Each participant functioned in a different social and home environment, which could have led to differences in the level of physical exertion, rehabilitation opportunities, and overall functional ability.

It should also be noted that the functional status of patients with heart failure, including those supported by an LVAD, depends on numerous clinical and non-clinical factors, which could not be fully standardized in the survey.

These findings should be interpreted in light of several methodological constraints, particularly the limited scope of available clinical data. The analysis did not include detailed parameters related to disease severity, laboratory and imaging findings, pharmacological treatment, or the course of therapy, all of which may influence both physical activity levels and functional status. Furthermore, physical activity was assessed based on participants’ self-reported responses, which may be subject to recall bias and subjective evaluation of individual behaviors. To standardize the analysis, participants were categorized as either physically active or inactive, without a detailed assessment of the intensity, frequency, and type of physical activity performed. Despite these limitations, the study provides valuable insights into the relationship between physical activity and functional status in patients with advanced heart failure, including those supported with a left ventricular assist device, and may serve as a basis for future research incorporating more comprehensive clinical data and objective methods of physical activity assessment.

## 5. Conclusions

In the present study, physically active patients with LVADs achieved significantly better functional outcomes compared to inactive individuals. Importantly, LVAD implantation alone, without maintaining regular physical activity, did not appear sufficient to improve functional capacity. These findings highlight the key role of physical activity as an essential component of rehabilitation in patients with advanced heart failure.

## Figures and Tables

**Table 1:** Inclusion and exclusion criteria applied in the study population selection process.

Inclusion Criteria	Exclusion Criteria
- adults aged >18 years old - a confirmed diagnosis of advanced heart failure corresponding to NYHA class III or IV - ongoing pharmacological management of advanced heart failure or prior implantation of a mechanical circulatory support device (LVAD) - voluntary and informed agreement to participate in the study	- aged under 18 years old - lack of a diagnosis of advanced heart failure classified as NYHA III or IV - absence of informed consent - inability to communicate effectively, preventing reliable assessment of quality of life

**Table 2:** Characteristics of participant groups.

		Group 1	Group 2	Group 3	Group 4
Age [years]	Mean	53.30	59.24	49.14	53.27
	Min	20	29	18	18
	Max	76	72	80	73
Sex	Women	24%	3%	42%	10%
	Men	76%	97%	58%	90%
Height [cm]	Mean	174.32	178.13	178.56	180.37
	Min	157	164	160	160
	Max	210	194	198	204
Smoking cigarettes	Yes	2%	9%	25%	10%
	No	69%	92%	75%	90%
Telemonitoring	Yes	2%	84%	3%	74%
	No	98%	16%	97%	26%
Weight [kg]	Mean	84.63	88.47	78.97	87
	Min	55	66	49	55
	Max	125	120	110	121
Place of residence	Village	28%	21%	14%	15%
	City with a population of up to 200,000 inhabitants	36%	35%	42%	43%
	City with a population of fewer than 200,000 inhabitants	35%	44%	44%	42%
Comorbidities	Yes	91%	89%	69%	73%
	No	9%	11%	31%	27%
Causes of heart failure	Most common	Ischemic cardiomyopathy (post-infarction)	Other	Ischemic cardiomyopathy (post-infarction)	Ischemic cardiomyopathy (post-infarction)
	%	46%	34%	71%	34%

**Table 3:** Comparison of SF-36 Physical Functioning and MLHFQ Physical Dimension scores across the study groups.

		Group 1	Group 2	Group 3	Group 4
SF-36	Mean [score]	16.7	22.2	19.1	23.4
	SD	4.7	4.2	5.8	5.2
MLHFQ	Mean [score]	24.4	14.4	18	10.6
	SD	9.7	7.1	10.2	9

**Table 4:** Spearman correlations between selected physical functioning items of the SF-36 questionnaire and MLHFQ scores.

Activity	ρ Value	Correlation Strength
Bending	ρ = −0.4	Moderate
Walking more than 1 km	ρ = −0.6	Strong
Walking approximately 500 m	ρ = −0.6	Strong
Walking approximately 100 m	ρ = −0.5	Strong
Running, weightlifting	ρ = −0.4	Moderate
Household activities such as dusting furniture and vacuuming	ρ = −0.6	Strong
Lifting and carrying groceries	ρ = −0.5	Strong
Climbing several flights of stairs	ρ = −0.6	Strong
Climbing one flight of stairs	ρ = −0.6	Strong
Bathing or dressing	ρ = −0.3	Moderate

**Table 5:** Correlations between selected MLHFQ items and SF-36 Physical Functioning scores.

Activity	ρ Value	Correlation Strength
Dyspnea	ρ = −0.6	Strong
Difficulty walking or climbing stairs	ρ = −0.6	Strong
Reduced social interaction	ρ = −0.6	Strong
Fatigue/lack of energy	ρ = −0.6	Strong
Sleep problems	ρ = −0.5	Strong
Limitations in household tasks	ρ = −0.5	Strong
Limitations in traveling	ρ = −0.5	Moderate
Need for rest during the day	ρ = −0.5	Moderate

## Data Availability

The original contributions presented in this study are included in the article. Further inquiries can be directed to the corresponding authors.

## Abbreviations

The following abbreviations are used in this manuscript:

LVAD	Left Ventricular Assist Device
SF-36	Short Form (36) Health Survey
MLHFQ	Minnesota Living with Heart Failure Questionnaire
HFeEF	HeRTf AILURE with Reduced Ejection Fraction
LVEF	Left Ventricular Ejection Fraction
HFmrEF	Heart Failure with Mildly Reduced Ejection Fraction
HFpEF	Heart Failure with Preserved Ejection Fraction
NYHA	New York Heart Association
ICD	Implantable Cardioverter Defibrillator
CRT	Cardiac Resynchronization Therapy
QRS	QRS Complex
ESC	European Society of Cardiology
EACTS	European Association for Cardio-Thoracic Surgery
KBV	Kassenärztliche Bundesvereinigung
MELD	Model for End-Stage Liver Disease
